# The gene drive bubble: New realities

**DOI:** 10.1371/journal.pgen.1006850

**Published:** 2017-07-20

**Authors:** James J. Bull, Harmit S. Malik

**Affiliations:** 1 Department of Integrative Biology, University of Texas at Austin, Austin, Texas, United States of America; 2 Division of Basic Sciences, Fred Hutchinson Cancer Research Center, Seattle, Washington, United States of America; 3 Howard Hughes Medical Institute, Fred Hutchinson Cancer Research Center, Seattle, Washington, United States of America; The University of North Carolina at Chapel Hill, UNITED STATES

“Life finds a way.”—Michael Crichton, Jurassic Park

The last couple of years have seen a profound rise in excitement about the many possible uses of gene drive systems (GDSs)—and of the possible drawbacks of this technology. Much of the focus has been on bioethical and biomedical questions about their implementation. On the plus side, a GDS could be used to suppress populations of disease vectors and invasive species. On the negative side, escape of a GDS into a beneficial species could spell its doom. So, how foolproof are they? That’s the question Champer and colleagues set out to answer in this issue of *PLOS Genetics*.

The potential use of GDSs for population control or disease suppression has been appreciated for nearly 60 years [1, 2]. Those possibilities were recognized in response to the discovery of natural forms of gene drive in mice and fruit flies [3–5]. Elementary theory was developed that showed the tremendous selective advantage of a “perfect” GDS, and it was easy to see that a lethal GDS could suppress or even wipe out a species [6–8]. Then, Austin Burt [9] suggested it would be possible to engineer GDSs to achieve these effects—but that was all theoretical. In spite of some exciting results with homing endonucleases [10, 11], an easy-to-engineer GDS remained an exciting but somewhat remote possibility.

**Fig 1:**
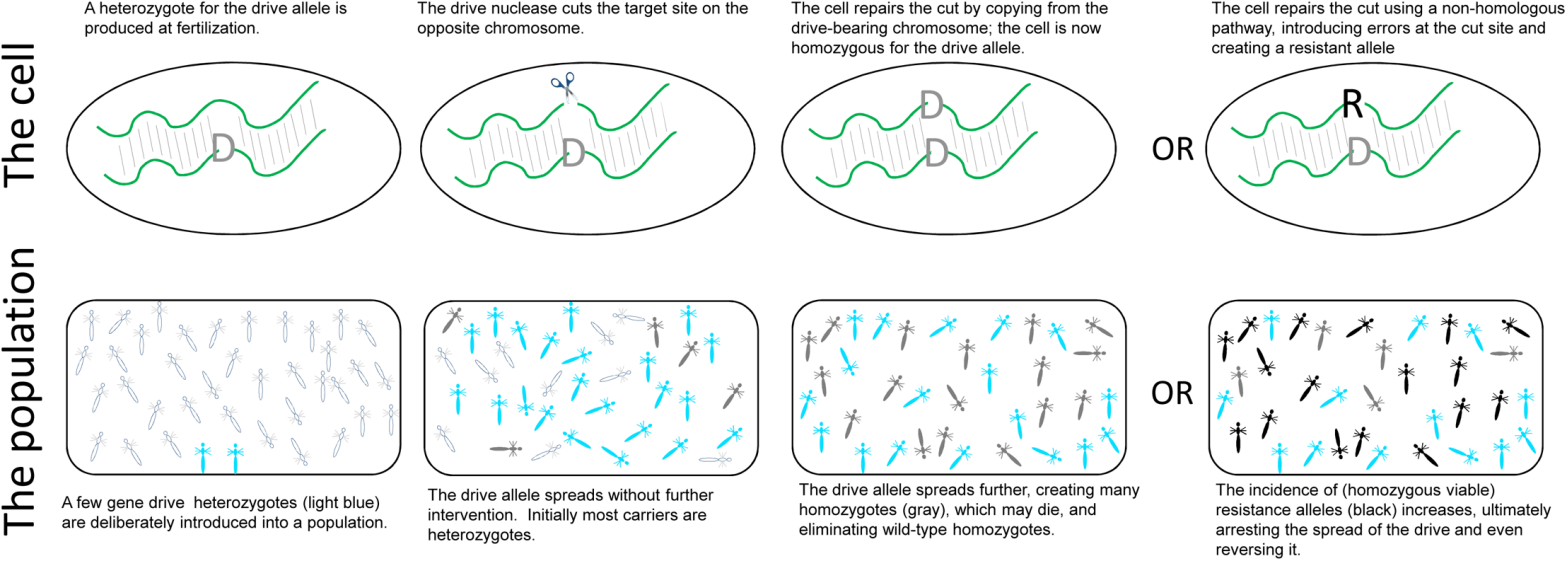
Gene drive systems (GDSs). (Top) GDSs consist of an endonuclease that cuts a specific region of the chromosome. When those nucleases are engineered into the very site that they cut, a cell that is heterozygous for the drive allele will experience a cut on the opposite, wild-type chromosome. The cell will repair the cut by copying from the intact (drive-bearing) chromosome, converting the former heterozygote into a homozygote for the drive allele. However, as an alternative to homology-based repair, the cell may use error-prone nonhomologous mechanisms to make a resistant allele that is not susceptible to the endonuclease. It is the very action of the nuclease that increases the likelihood of generating a resistant allele. (Bottom) This doubling of the drive allele in heterozygotes, if invariant and not deleterious to heterozygotes, provides a powerful evolutionary advantage. Clear individuals are wild-type homozygotes, light blue are drive heterozygotes, and gray are drive homozygotes. Introduction of just a few heterozygotes into a purely wild-type population will result in the drive allele sweeping through the population. Even when drive homozygotes die—lethal gene drive—the drive allele sweeps until everyone in the final population carries the drive allele. If the drive operates in both sexes, the final population is entirely homozygous and dead. If the drive operates in just 1 sex, the final population is a state in which half the progeny are heterozygotes, whereas the other half are homozygotes and die. Introduction of resistance alleles, particularly those that are homozygous viable (black), will impede and even reverse the spread of gene drive alleles.

All that changed thanks to the discovery of a bacterial nuclease system known as CRISPR-Cas9. The discovery of CRISPR-Cas9 meant that Burt’s proposal was closer to reality via the creation of a self-perpetuating gene drive that could be deployed in potentially any genomic location in any eukaryotic species [12]. The advantage of CRISPR-Cas9 over other known nucleases was that the Cas9 nuclease could be directed to cut almost any DNA sequence with exceedingly high specificity—Cas9 uses an easily designed guide RNA to find its target. A GDS is then easily created by inserting CRISPR-Cas9 into the very chromosomal location that it cuts; when the diploid cell is a heterozygote with a CRISPR-Cas9 construct on 1 chromosome but not on the other, CRISPR-Cas9 cuts the “empty” chromosome site, and the cell repairs the cut ends by copying from the CRISPR-Cas9–bearing chromosome (Fig 1). The formerly heterozygous cell now carries CRISPR-Cas9 on both chromosomes. If the cell is a germ cell, the heterozygote breeds as a homozygote, producing only CRISPR-Cas9–bearing gametes.

The selective advantage provided by this transmission “doubling” in heterozygotes is powerful (Fig 1). A CRISPR-Cas9 drive can be engineered into a somatically essential gene, provided that heterozygotes maintain normal fitness. Homozygotes will die, of course, and intuition would suggest that such engineering would be an evolutionary nonstarter because the spread of the drive allele should be quickly halted by homozygote death. However, the transmission advantage of drive in heterozygotes overwhelms the disadvantage of homozygote death, and if drive only operates in the germ line, the drive can spread despite homozygote death, even going to fixation if heterozygote conversion is 100% [6, 7]. If the drive activity is limited to 1 sex (e.g., only males experience the drive), the eventual outcome is a population in which 50% of zygotes die; if the drive operates in both sexes, ultimately all zygotes die and the population goes extinct. The extent of population suppression is sensitive to any imperfections in drive efficacy, but the effects are nonetheless impressive, even with imperfections [13]. GDSs can also be used to destroy nonessential genes without suppressing populations; the destruction of a suitably chosen nonessential gene might prevent a vector species from transmitting a pathogen or might render an otherwise resistant crop pest susceptible to a pesticide. But if the designed gene drive jumps into an unintended host, it can do all the damage that we hoped to impart on the intended target, and stopping a GDS once it is released is not easy—maybe not possible.

All of this hope and hype (and a little wariness) rests on the absence of easy resistance to GDSs. It has been apparent since the early discoveries of natural GDSs that alleles blocking the effect of a gene drive are favored under a broad range of conditions; more thorough theoretical work has reinforced those concerns [14, 15]. If the gene drive imparts a reduction in fitness at the population level (as with a GDS that suppresses the population), resistance that blocks the drive can be favored at virtually any location in the genome, meaning that the possibilities for resistance have a huge numerical advantage over the drive [16]. Indeed, Lyttle's caged populations of *Drosophila*—the first gene drive experiment to test population extinction—revealed at least 2 evolutionary pathways to resistance that allowed even his small populations to escape [17–19]. The evolution of resistance will in turn halt the spread of the gene drive, even reversing it, and any hoped-for outcome will be thwarted.

The easy targeting of CRISPR, the very property that has led to its current popularity, may also be its downfall as a practical means to control populations or suppress disease transmission. Simple changes in the target sequence can block the CRISPR nuclease, in turn reducing the rate at which heterozygotes become homozygotes. Champer et al. [20] show that resistance to a CRISPR gene drive engineered in the germ line of *D*. *melanogaster* can arise rapidly, often in a single generation. Moreover, through a simple but clever genetic design, the authors of the study discovered at least 2 different stages at which resistance arises: prior to fertilization in the germ line and after fertilization in the embryo. Importantly, this resistance is arising independently of direct selection, as the targeted gene *yellow* is not essential for viability. Rather, the resistance that is being scored is an approximate measure of a de facto mutation rate of drive-resistant alleles. Analysis of several resistant alleles suggests that they arose as a direct result of Cas9 action; however, instead of being genes converted to propagate the drive, they were simply “misrepaired” into resistance alleles. Thus, a potential Achilles’ heel of the self-perpetuating gene drives is the propensity of host genomes to favor error-prone DNA repair (leading to resistance) over conversion (leading to drive propagation). Indeed, Champer et al. find significant variation among different genetic backgrounds of *D*. *melanogaster* in terms of their propensity to acquire resistance. Of course, resistance rates may prove species specific or site specific, but even a 10-fold reduction in what is observed by Champer et al. is still discouragingly high. This “resistance” outcome would easily thwart virtually any intended application of a gene drive, and it poses a serious challenge to the many hoped-for applications of this technology.

One softening of this bad news is that the gene drive target sites used by Champer et al. are in nonessential genes. To be favored against a “lethal” GDS, resistance alleles need to be viable at a minimum. A nonessential target means that virtually any change in the sequence of the target site will generate a viable resistance allele. In contrast, a target site in an essential gene with highly conserved sequences will be less tolerant of sequence change, whereby many otherwise resistant alleles will be recessive lethals and thus will not evolve to block the gene drive. Even so, it is difficult to imagine that target sites are completely intolerant to change—a speculation supported by a new flour beetle study analyzing preexisting DNA sequence variation in genomic sites that would be prime candidates for CRISPR-Cas9 GDSs [21]. Considerable effort may be required to screen multiple target sites to find those that are least tolerant of change, and the use of highly conserved sequences as target sites may predispose engineered GDSs to jump into other species.

Understanding the evolution of resistance to gene drives may be the next phase in pursuit of this technology. To date, resistance has been acknowledged as possible but without empirical evidence of the magnitude of the problem. Burt [9] suggested 1 solution: introduce multiple gene drive constructs simultaneously at multiple locations in the genome. But even this strategy rests on resistance arising infrequently, which does not appear to be the case in the new study. Also, resistance need not be confined to changes in the target sequence. MicroRNAs and proteins that interfere with the CRISPR machinery can be favored and will block CRISPR gene drives no matter what the target sequence. Furthermore, a GDS derives its benefit from heterozygotes; highly structured or inbred populations are an impediment to gene drive spread, and inbreeding can actually evolve in response to a lethal gene drive [22]. Therefore, there is much yet to be done in bringing engineered gene drives to fruition. However, the potential rewards may justify the effort if the ecological and evolutionary benefits warrant their deployment.
